# Corticosteroid Use for Paradoxical Reactions during Antibiotic Treatment for *Mycobacterium ulcerans*

**DOI:** 10.1371/journal.pntd.0001767

**Published:** 2012-09-27

**Authors:** N. Deborah Friedman, Anthony H. McDonald, Michael E. Robson, Daniel P. O'Brien

**Affiliations:** 1 Department of Infectious Diseases, Barwon Health, Geelong, Victoria, Australia; 2 Department of Plastic Surgery, Barwon Health, Geelong, Victoria, Australia; 3 Saint John of God Pathology, Pathcare, Geelong, Victoria, Australia; 4 Department of Medicine and Infectious Diseases, Royal Melbourne Hospital, University of Melbourne, Victoria, Australia; Kwame Nkrumah University of Science and Technology (KNUST) School of Medical Sciences, Ghana

Buruli or Bairnsdale Ulcer (BU) is a neglected infectious disease caused by *Mycobacterium ulcerans* and is characterized by necrotic cutaneous lesions. Infection is challenging to treat, and the ideal combination of surgery and antimicrobial therapy continues to evolve. *M. ulcerans* has been endemic to the Bellarine peninsula in Victoria, Australia, since 1998, with more than 250 cases of infection. Studies have illustrated the safety and efficacy of antimicrobial therapy [1]–[5], and our standard treatment practice has evolved over the last 15 years to comprise limited surgical debridement with combination antimicrobial therapy.

Immune reconstitution inflammatory syndrome (IRIS) is a paradoxical reaction occurring during treatment of an infection, recognized clinically by deterioration after initial improvement. These reactions are well described in tuberculosis and leprosy, where effective antimicrobial killing may be accompanied by (transient) clinical deterioration during treatment [6], [7], predominantly in HIV-infected patients after the introduction of antiretroviral therapy [8]. IRIS reactions may also occur in patients with a competent immune system [9]. Our group and others have described paradoxical reactions occurring during the treatment of *M. ulcerans* infection with antimicrobials [10], [11].

In cases of *Mycobacterium tuberculosis* (TB) infection complicated by IRIS, steroid therapy is recommended. A randomized placebo-controlled trial of IRIS in TB found that prednisone reduced the need for hospitalization and therapeutic procedures and hastened improvements in symptoms, performance, and quality of life [12]. In our group's description of paradoxical reactions during therapy for BU, we proposed that adjunctive corticosteroid therapy may improve healing and prevent the need for further surgical intervention [10]. In the last 2 years our group has therefore acquired an early experience with the use of steroid therapy to treat severe IRIS in patients with *M. ulcerans* infection.

From 1998 through the end of 2011, our group has treated 163 patients with *M. ulcerans* infection with antimicrobials. We have assessed both retrospectively (until 2009) and prospectively (since 2009) that 31 patients (19%) developed paradoxical reactions. To date, five patients have been treated with steroid therapy for severe paradoxical reactions.

The patients treated with corticosteroids were aged between 9 and 84 years. All patients were ambulatory and managed as outpatients. These patients developed IRIS 2–13 weeks after commencing combination antimicrobial therapy after experiencing an initial clinical improvement in the erythema and induration surrounding the lesion. The clinical findings that were indicative of a severe paradoxical reaction included markedly increased inflammation and induration surrounding the *M. ulcerans* lesion, copious wound discharge, the appearance of new secondary lesions, and necrotic eschar formation (see Table 1).

**Table 1:** Clinical details of patients with paradoxical reactions treated with corticosteroids.

Age	Gender	Lesion Location	Time to Paradoxical Reaction (Days)	Initial Size of Lesion	Features of Paradoxical Reaction	Features after Steroid Therapy
62	F	Achilles region, ulcer	21	2×5 cm ulcer; induration 13×4 cm	Increased wound discharge and pain	Ulcer unchanged; discharge and pain resolved
83	F	Posterior heel, ulcer	92	3×3 cm ulcer; induration 5×8 cm	8×4 cm induration and wound ooze	2×2 cm lesion; no induration
9	F	Lower leg, ulcer	53	1 cm ulcer; minimal induration	Marked induration and wound discharge	Ulcer unchanged; swelling and discharge resolved
14	F	Knee, ulcer	17	8 mm ulcer; induration 2×2 cm	Induration 6×6 cm and wound discharge	Ulcer unchanged; induration and discharge resolved
84	M	Elbow, edematous lesion	30	No ulcer; induration 13×11 cm	Induration 28×20 cm	Induration resolved

Time to paradoxical reaction was defined as the number of days from the commencement of antibiotics until the onset of the paradoxical reaction.

**Figure 1:**
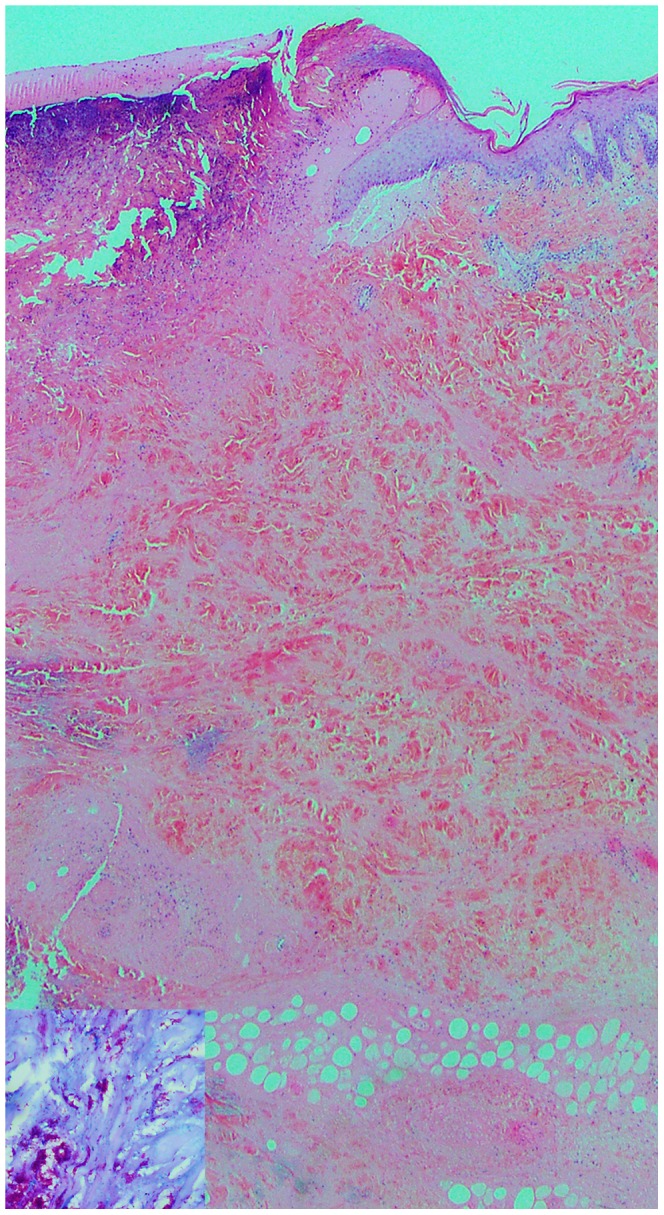
Skin biopsy from patient A after diagnosis of BU. Skin biopsy (×2.5 magnification) showing ulceration and extensive undermining necrosis of the dermis and subcutaneous fat with minimal inflammation, typical of B.U. Insert, Wade Fite stain (×40 magnification) showing numerous acid fast bacilli.

**Figure 2:**
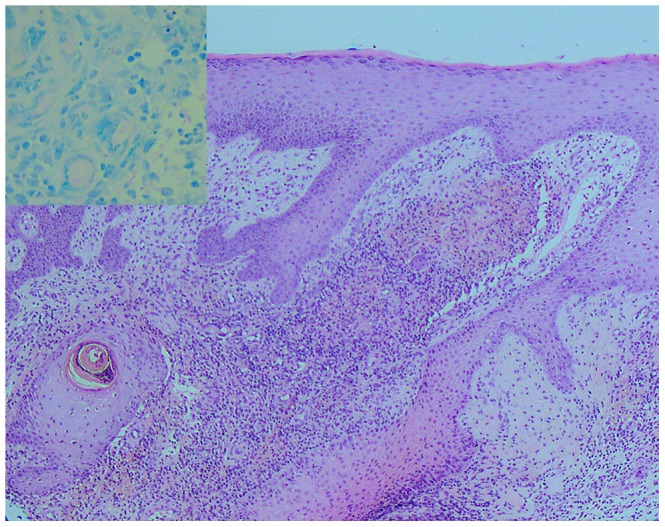
Skin biopsy from patient A with IRIS 7 weeks through antibiotic treatment for BU. Skin biopsy (×10 magnification) showing mixed inflammatory and granulation tissue reaction with prominent multinucleated giant cells, typical of a paradoxical reaction during antibiotic therapy for BU. Insert, Wade Fite stain (×40 magnification) showing very sparse acid fast bacilli.

Clinical deterioration during antibiotic treatment may be interpreted as treatment failure, leading to further potentially disfiguring and unnecessary surgery and either a change or prolongation of antimicrobial therapy [10]. Therefore, we believe that confirmation of a paradoxical reaction via histopathological assessment of a biopsy specimen and mycobacterial cultures are important in the therapeutic decision-making process for patients with clinical deterioration during antimicrobial therapy for *M. ulcerans*. Histopathological findings of a paradoxical reaction include ulceration and necrosis, a florid mixed inflammatory infiltrate with multinucleated giant cells, usually sparse or absent acid fast bacilli (AFB), and negative mycobacterial cultures (see Figures 1 and 2) [10], [13], although excised tissue may remain positive for *M. ulcerans* via polymerase chain reaction. In all of our cases with a paradoxical reaction, mycobacterial cultures were negative regardless of whether AFBs were seen on microscopy. We recognize that biopsy and histopathology may not be readily available in resource-limited settings managing patients with BU. In these cases, a diagnosis of paradoxical reaction based on clinical parameters will be most appropriate.

In our patients a provisional diagnosis of severe IRIS was followed by commencement of prednisone at a dose of 0.5–1 mg/kg daily with the aim of reducing further tissue destruction, preserving skin grafts, and limiting the extent of further surgery. In all patients with *M. ulcerans* treated with steroid therapy, there was a marked clinical improvement in the appearance of the lesion within days to weeks, and the duration of prednisone therapy used was 4–6 weeks, with gradual tapering of the dose after the first 2–3 weeks of therapy.

All patients tolerated prednisone well with no side-effects, and antimicrobial therapy was not changed nor prolonged beyond 12 weeks despite the occurrence of a paradoxical reaction. All patients underwent debridement of their *M. ulcerans* lesions before or after the commencement of the prednisone. Ultimately all have achieved complete healing without recurrence between 9–21 months after initial treatment.

*M. ulcerans* produces a necrotizing macrolide toxin, mycolactone, which profoundly suppresses elements of innate and adaptive cell-mediated immunity, thereby enhancing progression of BU [14]. Histopathologically, BU lesions are characterized by a poor inflammatory response. In some BU lesions, treatment with antibiotics may cause temporary immune-mediated inflammation with clinical worsening, which we and others have proposed as a paradoxical sign of treatment success [10], [11].

In the cases that we have treated with prednisone for a paradoxical reaction during therapy for *M. ulcerans* infection, subsequent severe clinical deterioration has occurred after initial improvement on antibiotic therapy. This may be explained by an apparent reversal of the local immune-tolerant state of active *M. ulcerans* infection due to antibiotic therapy. A local cellular immune response has been shown to develop in these situations as production of mycolactone is reduced [14], presumably in response to persisting mycobacterial antigens. We believe that in our patients, the current practice of limited surgical debridement with combination antimicrobial therapy results in incomplete excision of mycobacteria-infected tissue, which may potentiate the development of paradoxical reactions.

As with paradoxical immune reactions to antimicrobial therapy for other mycobacterial diseases where corticosteroids are known to be effective [12], [15], our approach with these BU infections was to commence prednisone as adjunctive treatment to antibiotics to settle the severe immune-mediated reaction and limit secondary tissue damage. The dose was chosen based on recommendations for the treatment of TB-IRIS reactions [12]. The use of prednisone in these BU cases was associated with a settling of the skin lesions and ultimately prevented the need for further extensive surgery. Furthermore, complete healing occurred despite not extending the duration of antimicrobial therapy beyond 12 weeks.

We acknowledge several factors that may limit the use of steroid therapy in this clinical setting. Prednisone may adversely affect outcomes by suppressing the host immune response to *M. Ulcerans*, and reduced serum levels of prednisone may result from an interaction with rifampin [16], [17]. In addition, the use of steroid therapy is associated with many potential complications, and therefore, not all patients with BU may be optimal candidates for steroid therapy. For example, in BU endemic areas with high rates of co-existent infections such as tuberculosis or strongyloidiasis, prednisone may adversely affect the outcomes of these co-infections [18]. It is advisable during therapy with corticosteroids that the following parameters be monitored: mood changes, sleep disturbance, and increase in appetite. Blood glucose levels and blood pressure should be monitored where there is a history of diabetes mellitus or hypertension or a clinical predisposition to either of these conditions.

There are potential limitations to our purely observational description of success with the use of adjunctive steroid therapy in patients with a severe paradoxical reaction during antimicrobial treatment for BU. For instance, in the absence of a control group, it is unclear what the progress of the healing of these skin lesions would have been without steroid therapy, and whether the outcome may have been the same. Furthermore, the use of limited debridement of lesions in conjunction with antimicrobials, both of which are known to be effective in the cure of *M. ulcerans* infection [1]–[3], may have influenced healing significantly such that the effect of steroids alone is unclear. Nonetheless, there is historical precedence that is supportive of corticosteroid use in paradoxical reactions with other mycobacterial infections [12].

To our knowledge this is the first description of corticosteroid use in cases of *M. ulcerans* where severe paradoxical reactions to antibiotic treatment have occurred. In our early experience, the use of prednisone at a dose of 0.5–1 mg/kg was clinically effective and well tolerated. Moreover, the use of steroid therapy in addition to antimicrobial therapy in our patients was associated with good cosmetic outcomes, no significant adverse effects, and no need for further major surgical intervention. Hence we would advocate for further prospective study of corticosteroid use in the treatment of *M. ulcerans*–associated severe paradoxical reactions.
